# M. Kun’s New Instrument for the Diagnosis of Tumors

**Published:** 1848-05

**Authors:** 


*M. Kün's New Instrument for the Diagnosis of Tumors.*—M. Kün, Professor of Physiology in Strasbourg, presented to the Medical Society of that city, an instrument, the application of which is likely to produce the most beneficial results in the diagnosis of various kinds of tumor. It consists of an exploring needle, having at its extremity a small depression with cutting edges. On plunging this instrument into a tumor to any depth, we can extract a minute portion of the tissue of which its various layers are composed. In this manner a microscopic examination of the tumor can be practised on the living subject, and its nature ascertained before having recourse to an operation. We have proved the utility of this method of diagnosis on three occasions, and seen conscientious practitioners renounce an operation previously deter mined on when the cancerous nature of the tumor has been demonstrated by the microscope.—*Jour. Med. Science.*—*Ibid.*

